# Extracting Drug Names and Associated Attributes From Discharge Summaries: Text Mining Study

**DOI:** 10.2196/24678

**Published:** 2021-05-05

**Authors:** Ghada Alfattni, Maksim Belousov, Niels Peek, Goran Nenadic

**Affiliations:** 1 Department of Computer Science University of Manchester Manchester United Kingdom; 2 Department of Computer Science Jamoum University College Umm Al-Qura University Makkah Saudi Arabia; 3 Centre for Health Informatics Division of Informatics, Imaging and Data Sciences University of Manchester Manchester United Kingdom; 4 National Institute of Health Research Manchester Biomedical Research Centre Manchester Academic Health Science Centre University of Manchester Manchester United Kingdom; 5 The Alan Turing Institute Manchester United Kingdom

**Keywords:** information extraction, electronic health records, discharge summaries, natural language processing, medication prescriptions

## Abstract

**Background:**

Drug prescriptions are often recorded in free-text clinical narratives; making this information available in a structured form is important to support many health-related tasks. Although several natural language processing (NLP) methods have been proposed to extract such information, many challenges remain.

**Objective:**

This study evaluates the feasibility of using NLP and deep learning approaches for extracting and linking drug names and associated attributes identified in clinical free-text notes and presents an extensive error analysis of different methods. This study initiated with the participation in the 2018 National NLP Clinical Challenges (n2c2) shared task on adverse drug events and medication extraction.

**Methods:**

The proposed system (DrugEx) consists of a named entity recognizer (NER) to identify drugs and associated attributes and a relation extraction (RE) method to identify the relations between them. For NER, we explored deep learning-based approaches (ie, bidirectional long-short term memory with conditional random fields [BiLSTM-CRFs]) with various embeddings (ie, word embedding, character embedding [CE], and semantic-feature embedding) to investigate how different embeddings influence the performance. A rule-based method was implemented for RE and compared with a context-aware long-short term memory (LSTM) model. The methods were trained and evaluated using the 2018 n2c2 shared task data.

**Results:**

The experiments showed that the best model (BiLSTM-CRFs with pretrained word embeddings [PWE] and CE) achieved lenient micro F-scores of 0.921 for NER, 0.927 for RE, and 0.855 for the end-to-end system. NER, which relies on the pretrained word and semantic embeddings, performed better on most individual entity types, but NER with PWE and CE had the highest classification efficiency among the proposed approaches. Extracting relations using the rule-based method achieved higher accuracy than the context-aware LSTM for most relations. Interestingly, the LSTM model performed notably better in the reason-drug relations, the most challenging relation type.

**Conclusions:**

The proposed end-to-end system achieved encouraging results and demonstrated the feasibility of using deep learning methods to extract medication information from free-text data.

## Introduction

### Background

Electronic health records (EHRs) are a valuable source of routinely collected health data that can be used for secondary purposes, including clinical and epidemiological research [1]. They typically contain information on consultations, admissions, symptoms, clinical examinations, test results, diagnoses, treatments, and outcomes. Medication prescriptions are a key source for understanding the effects of patient treatment. In some settings (eg, general practitioners’ practices), they might be recorded in a structured fashion through prescribing software and would comprise, apart from drug names, medication attributes such as dosage, frequency, and duration. Still, there are often additional, free-text sources of prescription information, such as clinic letters or discharge summaries, particularly in secondary care. Extracting information from free-text is challenging because much of the information is provided in a narrative manner, and the text is often written in haste and under considerable time pressure. There has been strong interest among researchers in the use of natural language processing (NLP) to extract information from clinical free-text notes on a large scale [2-9], including a number of shared tasks and benchmark data sets to assess and advance the state-of-the-art in this domain, such as challenges in medication extraction [7]; chemical and drug named entity recognition (NER) [10]; drug-drug interaction extraction [11]; and extraction of medications, indications, and adverse drug events (ADEs) [12,13].

Medication prescription instructions are a specific clinical sublanguage, where expressions are often abbreviated (eg, *od* for *once a day*) and may contain spelling errors (eg, *20 mcg evry othr wk*) [14,15]. Existing approaches for extracting drugs and associated attributes from the clinical text are diverse in their methods, using various approaches including dictionary lookup (ie, searching for matches from existing drug dictionaries) [16-18], rule-based approaches (manually design patterns, eg, regular expressions that can be searched in free-text) [2-4,8,14,16,19-22], machine learning approaches (training models on example data) [23-28], and hybrid approaches that combine different methods [29-32]. Recently, methods based on deep learning and neural networks, such as convolutional neural networks and recurrent neural networks, have been shown to be state-of-the-art in drug attribute extraction tasks [33-41]. Deep learning methods take relevant features (eg, orthographic and lexical features) as inputs and produce labels as outputs. These manually constructed feature vectors can then be replaced with, for example, word embeddings (WE), character embeddings (CEs), and feature embeddings. Embeddings are representations of tokens in an n-dimensional space, typically learned over large collections of unlabeled data through an unsupervised process (eg, word2vec [42], Global Vectors for Word (GloVe) [43], and fastText [44]). Recently, more advanced embedding methods and representations (eg, Embeddings from Language Models [ELMo] [45] and Bidirectional Encoder Representations from Transformers [BERT] [46]) have further advanced state-of-the-art clinical NLP.

### Objectives

Although deep learning methods have been extensively used in medication information extraction [13], the effects of various architectures and token representations have not been widely discussed. The purpose of this study is to provide a comprehensive comparison of various representations used for drug information extraction within the same settings. The main contributions of our work are as follows:

- An investigation of the effect of various token representations (ie, CE, WE, and semantic-feature embeddings [SFEs]) on extracting medication information
- The comparison between a rule-based method and deep learning approaches for identifying relations between drugs and associated attributes.

## Methods

### Overview

The DrugEx system proposed here is composed of (1) an NER method for extracting mentions of drug names and drug-associated attributes and (2) a relation extraction (RE) method for identifying relations between drugs and their associated attributes. The NER task involves extracting 8 types of entities: drug, strength, duration, route, form, dosage, frequency, and reason of administration (see Textbox 1 for definitions and examples of the extracted entities).

**Definitions and examples of entity types extracted by the DrugEx system.**

- Drug: The chemical name of a drug or the advertised brand name under which a drug is sold (eg, aspirin)
- Dosage: The amount of medicine that the patient takes or should take (eg, 2 tablets, 5 mL)
- Strength: The amount of drug in a given dosage (eg, 200 mg)
- Frequency: The rate at which medication was taken or is repeated over a particular period (eg, daily, every 4 hours)
- Duration: The period of continuous medication taking (eg, *pro re nata*, for 5 days)
- Route: The path by which medication is taken into the body or the location at which it is applied (eg, topical, *per os*)
- Form: The form in which a medication is marketed for use (eg, tablet)
- Reason: The reason for medication administration (eg, for pain)

The scope of these entity types and the data sets that were used for training and evaluation were provided as part of the 2018 National NLP Clinical Challenges (n2c2) shared task track 2: ADEs and medication extraction in EHR challenge [13,47]. The data set consists of discharge summaries drawn from the Medical Information Mart for Intensive Care III (MIMIC-III) clinical care database [48]. It comprises 505 documents, of which 303 documents were used as the training set, and the remaining 202 documents were used as the test set. These data were annotated by 7 domain experts, consisting of 4 physician-assistant students and 3 nurses. Annotations included drug, strength, dosage, frequency, duration, form, route, reason, and ADEs; ADEs annotations have been omitted here as they are beyond the scope of this study.

The annotations also included relations between drugs and other attributes. Table 1 shows the descriptive statistics for the associated drug attributes in the n2c2 data set and how often each of them was linked to more than 1 drug. Noticeably, 17% (1412/8579) of the reason entities were associated with more than one drug; the maximum number of drugs associated with a single reason was 10. For example, in “START: Guaifensin with codeine QHS and Benzonatate as needed for cough,” the reason *cough* is associated with 2 drugs: guaifenesin (with codeine) and benzonatate. Table 2 shows the number of drug entities participating in each link and the ratio of drugs with more than one link. From a total of 11,028 form-drug relations, 4517 (41%) drugs that have been associated with the form attribute has more than one association (ie, multiple forms reported for a single drug entity), for example, “Bisacodyl 5 mg Tablet Sig: 1-2 Tablets PO once a day as needed for constipation;” both mentions of tablets were annotated as form, and they both associated to the bisacodyl drug.

**Table 1:** Descriptive statistics of entity types in the National NLP Clinical Challenges (n2c2) data set.

Entity types	Entities, n (%)	Links to 1 drug, n (%)	Links to multiple drugs, n (%)	Maximum number of drug associations
Drug	26,800 (32.57)	—^a^	—	—
Form	11,010 (13.38)	10,980 (99.56)	48 (<1)	2
Strength	10,921 (13.27)	10,913 (99.70)	33 (<1)	3
Frequency	10,293 (12.51)	10,281 (99.39)	63 (1)	4
Route	8989 (10.92)	9000 (99.08)	84 (1)	4
Dosage	6902 (8.39)	6877 (99.38)	43 (1)	4
Reason	6400 (7.78)	7158 (83.44)	1421 (16.56)	10
Duration	970 (1.2)	991 (92.7)	78 (7)	4

^a^Not applicable.

**Table 2:** Descriptive statistics of relations between drugs and their associated attributes in the National NLP Clinical Challenges (n2c2) data set.

Relation type	Relations, n (%)	Drugs with 1 link, n (%)	Drugs with more than 1 link, n (%)
Strength-drug	10,946 (18.88)	10,639 (97.20)	307 (2.8)
Frequency-drug	10,344 (17.84)	10,054(97.20)	290 (2.8)
Route-drug	9084 (15.67)	8903 (98.01)	181 (1.99)
Reason-drug	8579 (14.80)	7704 (89.80)	875 (10.2)
Dosage-drug	6920 (11.94)	6765 (97.76)	155 (2.2)
Form-drug	11,028 (19.02)	6511 (59.04)	4517 (40.96)
Duration-drug	1069 (1.84)	1021 (95.51)	48 (5)

### NER Method

**Figure 1:**
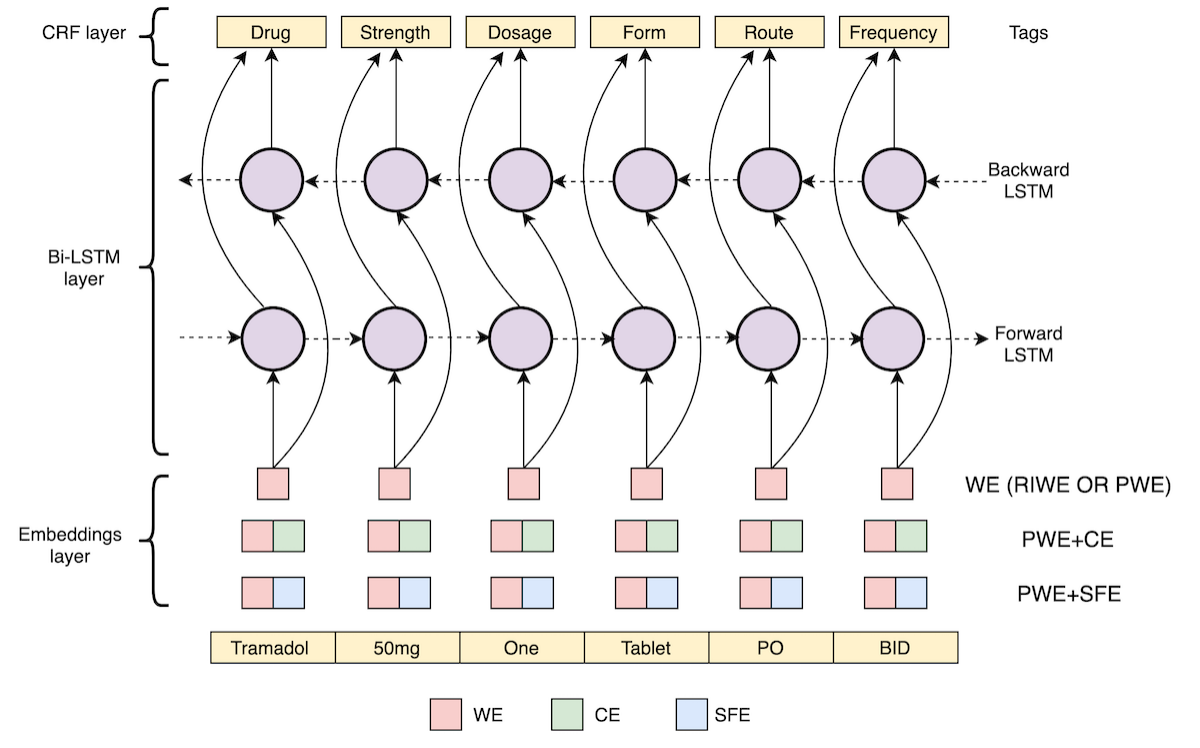
The architecture of bidirectional long-short term memory with conditional random field for the named entity recognition models. BiLSTM-CRF: bidirectional long-short term memory with conditional random field; PWE+CE: pretrained word embeddings and character embeddings; PWE: pretrained word embeddings; PWE+SFE: pretrained word embeddings and semantic-feature embeddings; RIWE: randomly initialized word embeddings; WE: word embeddings.

All NER models rely on bidirectional long-short term memory with conditional random fields (BiLSTM-CRF) architecture (Figure 1), which is composed of 3 different layers: embedding layer, bidirectional long-short term memory (BiLSTM) layer, and conditional random fields (CRFs) layer.

#### Preprocessing

The data were first tokenized using spaCy, an open-source library for NLP, with support for various languages. Then, as target entities differ in length and may contain more than one token, each token was annotated using the BIOES (Begin, Inside, Outside, End, Single) tagging scheme to capture information about the sequence of tokens. We further processed the discharge summaries using the Clinical Language Annotation, Modeling, and Processing Toolkit (CLAMP) [49] and the Clinical Text Analysis and Knowledge Extraction System (cTAKES) [50] to extract token-level clinical semantic tags (eg, medication, disease disorder, and procedure; see the section *Embedding Layer* for details), which were used for SFEs.

#### Embedding Layer

The embedding layer maps tokens into vectors of numbers that represent their meanings. WEs provide dense representations that make them capable of representing many aspects of similarities between words, such as semantic relations and morphological properties [51,52]. Several methods can be used to initialize the values in WEs at the beginning of neural network training. We examined the randomly initialized word embeddings (RIWE) and the pretrained word embeddings (PWE), where the latter has been pretrained on data from the clinical (ie, target) domain.

Although WEs can capture tokens’ semantics, they might still be affected by data sparsity and, therefore, cannot remediate synonyms, out-of-vocabulary tokens, and misspellings. WE may not be able to capture morphemes (such as prefixes and suffixes) derived from classic Latin and ancient Greek roots, which are often included in drug names and drug attributes. Thus, we addressed these issues by using character feature embeddings in addition to WEs. The concatenation of the PWE with the CEs allows the model to learn subtoken patterns such as morphemes and roots, thereby aiming to capture out-of-vocabulary tokens, different forms, and any other information not captured by WEs [53].

**Figure 2:**
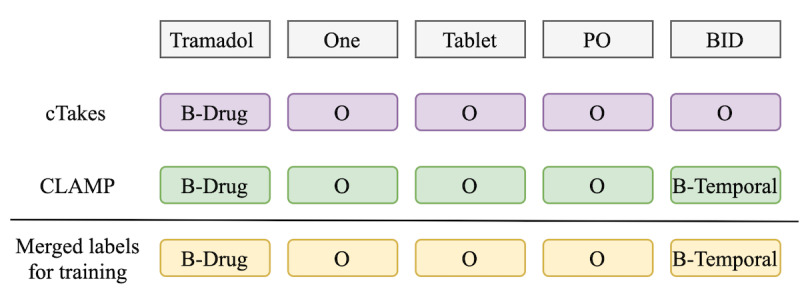
Semantic-feature token embeddings. B-Drug: begin-drug; B-Temporal: begin-temporal; CLAMP: Clinical Language Annotation, Modeling, and Processing Toolkit; cTakes: Clinical Text Analysis and Knowledge Extraction System; O: outside.

We also considered representations beyond tokens, aiming to add clinical semantics to words. Specifically, the concatenation of the PWE and SFEs was used to represent the clinical categories of entities identified in the text, such as medical problems, tests, or temporal information. Note that in this study, we did not evaluate SFE without PWEs. Some entity types (such as frequency or route) are not present among the semantic tags we used, whereas other semantic tags (such as signs, symptoms, disease, and disorder) are more frequent. Therefore, the representations of semantic tags were learned simultaneously with word representations and concatenated together to form the final token representations. We used CLAMP [49] to extract semantic tags (ie, problem, treatment, and temporal entities) with associated assertion tag attributes (ie, present or absent). We also used the default clinical pipelines in cTAKES [50] to tag tokens with other semantic categories (ie, Medication, DiseaseDisorder, and SignSymptom). In each pipeline, tokens were tagged with the corresponding semantic features and attributes (if available); otherwise, they were tagged with the outside (ie, O) tag. Token-level semantic tags from both pipelines were then mapped and merged based on their types to create a set of semantic features (Figure 2).

#### BiLSTM Layer

The BiLSTM layer takes the sequence of vectors (ie, token representations) corresponding to a sequence of tokens (the output from the embedding layer) and calculates the hidden states by processing the sequence of token representations forward and backward (ie, left-to-right and right-to-left) to learn important token-level features. It then outputs the sequence of vectors, including the probability of each label for each corresponding token. The labels were either 1 of the 8 entity types (Textbox 1) or none. The label assigned to the token is the label with the highest probability from the predicted labels’ sequence (output from the BiLSTM layer).

#### CRF Layer

The BiLSTM output does not consider the dependencies between neighboring labels when predicting the current label. For example, it may be more likely to have a token labeled as a drug name followed by a token labeled as *strength* than any other entity type. Thus, to learn these dependencies, we added a CRF layer that uses past and future labels to optimize predictions and obtain the most probable sequence of predicted labels. Finally, the labels (BIOES tags) were combined into named entities by merging consecutive labeled B-, I-, E-, or S-tags of the same class.

#### NER Models Training and Tuning of Hyperparameters

We used the standard data split established by the n2c2 organizers, using the training set for fitting models, tuning the model parameters, and evaluating our best models on the test set. As there is no official development set, we randomly selected 9.9% (30/303) of the training documents for validation. This data set was used to optimize the models’ hyperparameters.

We trained all neural network models using stochastic gradient descent, with a learning rate of 0.005. In the baseline model (RIWE), we randomly selected 100-dimensional WEs. In other models, we used pretrained 600-dimensional WEs [54], which were trained on approximately 2 million discharge summaries drawn from the MIMIC-III data [48] using the word2vec continuous bag-of-words method [42]. CEs were 25-dimensional vectors, whereas SFEs were 50-dimensional vectors. The number of hidden states was set to 300 dimensions for running the BiLSTM WEs and to 25-dimensions for running the BiLSTM for learning CE. We also applied dropout to the token embeddings at a rate of 0.5 to avoid overfitting. The number of epochs was determined by an early stopping criterion (ie, after 10 epochs with no improvement) on the validation set, with the maximum number of epochs set to 100. Finally, the batch size was set to 32. These hyperparameters were optimized through a random search of the validation set [55]. We tested WEs with dimensions ranging from 100 to 600, CE and SFEs with 25, 50, and 100 dimensions, and the dropout rate with values in the range between 0 and 0.75.

### RE Method

Once drugs and attributes are extracted, the subsequent step is to link drug names to the corresponding attributes. For this task, we experimented with a rule-based method engineered for the task and a context-aware long-short term memory (LSTM) model, where the positions of the involved entities were encoded using marker embeddings.

#### Context-Aware LSTM

**Figure 3:**
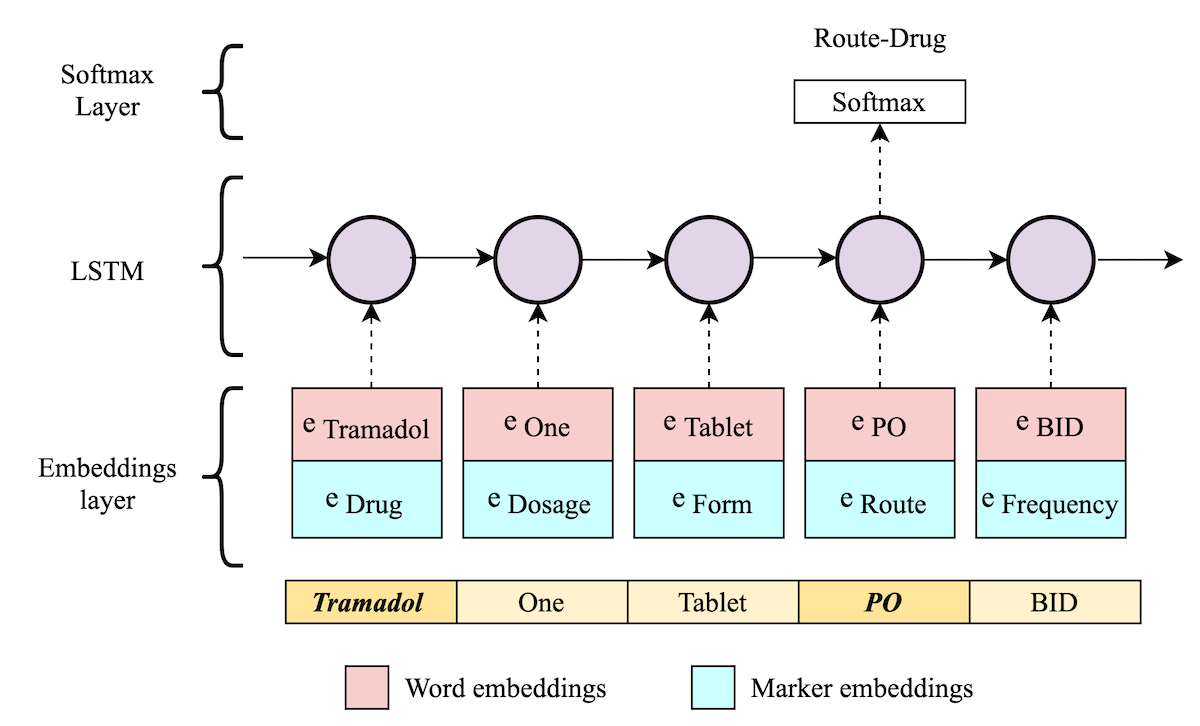
The architecture of context-aware long-short term memory for the relation extraction model. e: embedding; LSTM: long-short term memory.

We used a context-aware LSTM [56] that considers other relations in the sentential context while predicting the target relation. It uses an LSTM-based encoder to jointly learn representations for all relations in the text. Thus, the representation of the target relation and representations of the context relations are combined to make the final prediction. Figure 3 presents the architecture of the LSTM model for RE. It consists of an embedding layer, an LSTM layer, and a softmax layer. The embedding layer maps a portion of the text that contains a target entity pair into a high-level representation vector. First, each token in the text is mapped to its WE vector. Second, every 2 entities (ie, a drug and its associated attribute) in the text are paired as candidate entities for a possible relation. All other tokens are then marked as either belonging to a drug (as the main actor of all relations) or not. Afterward, each token’s marker embeddings are concatenated to the WEs to generate a single vector. This vector is then passed to the LSTM, which calculates the hidden states by processing the sequence of token representations. Finally, the LSTM layer’s output is routed into the softmax layer to map the nonnormalized output to the final output vector that contains the probability for each relation type.

#### Rule-Based Method

**Figure 4:**
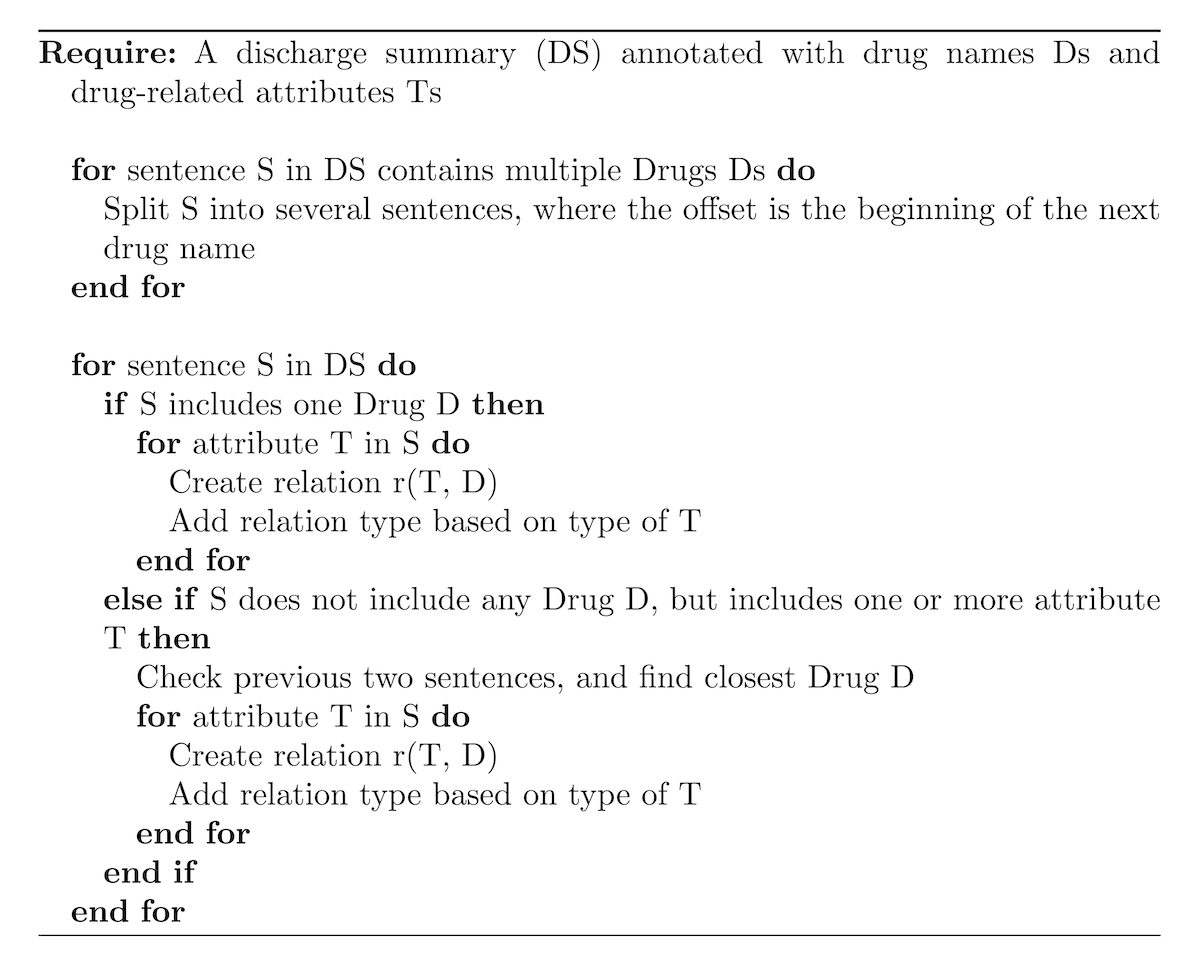
Rule-based method for linking drug names to corresponding attributes in discharge summaries.

In this approach, we examined patterns of prescription information in discharge summaries in the training set and manually implemented a set of rules using regular expressions. These regular expressions were designed and implemented in the General Architecture of Text Engineering environment [57] (Figure 4). First, the discharge summaries were split into sentences. For sentences that include only one drug name D, all drug attributes found in that sentence will be linked to drug D. However, for sentences that include multiple drug names, the sentences are split into several segments, where the segment’s start offset is the beginning of the next drug name.

If a sentence does not include a drug name but contains other entities, then the previous 2 sentences are checked. If they contain a drug name, then the attributes are linked to the closest drug name. For example, “Patient will be on Topiramate *25mg PO BID* until 22/3 PM. Then increase to *50mg po BID for seven days*. Then increase to *75mg ongoing*”. All the italicized entities are linked to the drug *topiramate* that appears in the first sentence.

#### RE Model Training and Tuning of Hyperparameters

We used the same procedure and the same approach for hyperparameter settings that we have used previously in the NER models. Specifically, we trained the LSTM model using the same hyperparameters that we have used previously in the NER models. We used marker embeddings with 10-dimensional vectors.

The regular expressions in the RE rule-based method were implemented based on manual observation of the training set, followed by an initial evaluation of the validation set. The regular expressions were then refined based on an error analysis of the output from the validation process, and the final evaluation was performed on the official test set.

### Evaluation

We considered the available annotations in the corpus as the gold standard when evaluating the models. To assess the performance of the proposed models, we performed hold-out cross-validation (using training and testing sets) and used the official n2c2 evaluation script provided with the data. It uses standard evaluation methods in information retrieval (ie, precision, recall, and F-score). We report the lenient micro-and macroaveraging for each NER experiment. Lenient matches refer to cases where the overlapped boundaries between the gold standard and the system’s predictions are allowed. Macroaveraging calculates the metrics on a per-document basis and then averages the results. Microaveraging, on the other hand, refers to the pooling of the results of all classified instances into a single contingency table.

In addition, we evaluated the performance of the NER models with the best-performing RE model as an end-to-end system. This allows us to measure the effect of missing entities in the NER models on the RE task. As shown in Table 1, attributes could be associated with more than one drug. Thus, when an NER model fails to recognize an entity (either drug or attribute), then all of its semantic relations (ie, associations) will also be missed. Finally, the best-performed end-to-end system was chosen for our DrugEX system.

## Results

### NER Task

Table 3 shows the lenient precision, recall, and F-score for all models in the NER task. The best result in the NER task was achieved by PWE+CE embeddings (micro F-score of 0.921). Interestingly, NER (PWE), which ranked second in F-score, achieved a slightly higher precision, and NER (PWE+SFE) achieved a higher recall than any other model. NER (PWE+SFE) also yielded a better balance between precision and recall. Concerning individual F-scores, PWE performed better than the baseline (RIWE) for every entity type. The SFEs with the PWEs in NER (PWE+SFE) allow the model to perform better than others on some individual entity types, especially frequency, duration, and reason. An analysis at the per-entity type level shows that most entity types (ie, drugs, strength, form, dosage, frequency, and route) are associated with excellent performance (F-scores above 0.90). Duration and reason, however, are associated with lower performance. This might be amplified by the fact that there were few examples of duration and reason entities in the training data (Table 1).

**Table 3:** Evaluation results of the named entity recognition models on the test set (lenient evaluation).

Entity	RIWE^a^				PWE^b^				(PWE+CE)^c^				(PWE+SFE)^d^		
	Precision	Recall	F-score	Precision		Recall	F-score	Precision		Recall	F-score	Precision		Recall	F-score
Drug	0.942	0.892	0.917	0.963		0.930	0.946	0.946		0.953	0.949	0.952		0.947	*0.950^e^*
Strength	0.977	0.959	0.968	0.979		0.970	0.975	0.973		0.976	0.974	0.977		0.977	*0.977*
Duration	0.893	0.706	0.789	0.883		0.762	0.818	0.910		0.698	0.790	0.903		0.786	*0.840*
Route	0.964	0.928	0.946	0.964		0.938	0.951	0.956		0.948	*0.952*	0.953		0.943	0.948
Form	0.964	0.935	0.949	0.965		0.940	0.952	0.969		0.944	*0.956*	0.972		0.932	0.951
Dosage	0.928	0.912	0.920	0.932		0.931	*0.931*	0.931		0.928	0.929	0.928		0.931	0.930
Frequency	0.945	0.925	0.935	0.965		0.952	0.959	0.980		0.933	0.956	0.968		0.968	*0.968*
Reason	0.771	0.458	0.575	0.821		0.497	0.620	0.860		0.452	0.593	0.621		0.653	*0.637*
Micro	0.943	0.863	0.901	0.951		0.892	0.921	0.950		0.894	*0.921*	0.927		0.913	0.920
Macro	0.936	0.840	0.883	0.951		0.876	0.910	0.949		0.884	*0.914*	0.923		0.901	0.910

^a^RIWE: bidirectional long-short term memory with conditional random fields with random word embeddings.

^b^PWE: bidirectional long-short term memory with conditional random fields with pretrained word embeddings.

^c^(PWE+CE): bidirectional long-short term memory with conditional random fields with pretrained word embeddings and character embeddings.

^d^(PWE+SFE): bidirectional long-short term memory with conditional random fields with pretrained word embeddings and semantic-feature embeddings.

^e^The best results for each metric are italicized.

To explore the complementarity of the methods, we created an ensemble model using the outputs of all the proposed NER models. The ensemble output for each task was generated using a majority voting scheme. In addition to its type, the entire named entity phrase is taken as 1 prediction instance. The ensemble model showed precision, recall, and F-scores of 0.961, 0.884, and 0.921, respectively. As expected, the ensemble showed performance gains in precision when compared with the best individual models. This indicates that the 3 models did not learn the same patterns from the data set. However, the difference in recall and F-score is not evident, even for specific attributes (Table 4).

**Table 4:** Evaluation results of pretrained word embeddings+character embedding named entity recognition model, pretrained word embeddings+character embedding named entity recognition model, and the ensemble model on the test set (lenient evaluation).

Entity	(PWE+CE)^a^				(PWE+SFE)^b^				Ensemble		
	Precision	Recall	F-score	Recall		Precision	F-score	Precision		Recall	F-score
Drug	0.946	0.953	0.949	0.952		0.947	*0.950^c^*	0.962		0.939	*0.950*
Strength	0.973	0.976	0.974	0.977		0.977	*0.977*	0.981		0.972	*0.977*
Duration	0.910	0.698	0.790	0.903		0.786	*0.840*	0.919		0.720	0.807
Route	0.956	0.948	0.952	0.953		0.943	0.948	0.963		0.944	*0.953*
Form	0.969	0.944	*0.956*	0.972		0.932	0.951	0.972		0.939	0.955
Dosage	0.931	0.928	0.929	0.928		0.931	0.930	0.943		0.930	*0.936*
Frequency	0.980	0.933	0.956	0.968		0.968	*0.968*	0.979		0.915	0.946
Reason	0.860	0.452	0.593	0.621		0.653	*0.637*	0.858		0.476	0.613
Micro	0.950	0.894	*0.921*	0.927		0.913	0.920	0.961		0.884	*0.921*
Macro	0.949	0.884	*0.914*	0.923		0.901	0.910	0.962		0.869	0.911

^a^(PWE+CE): bidirectional long-short term memory with conditional random fields with pretrained word embeddings and character embeddings.

^b^(PWE+SFE): bidirectional long-short term memory with conditional random fields with pretrained word embeddings and semantic-feature embeddings.

^c^The best results for each metric are italicized.

We further conducted paired *t* tests to determine whether the differences between the models were statistically significant. Differences were considered significant if the *P* value was <.05. The samples used in this test were the microaverage F-scores from each document in the test set (ie, document-level NER performance). Table 5 shows the post hoc analysis of variance for the NER task. The statistical significance test showed that there were no statistically significant differences between any of the models (PWE, PWE+CE, and PWE+SFE), despite the presence of apparently important and computationally expensive clinical information such as the type of entities (ie, problems, signs, and symptoms) in some of the models. However, the 3 models (PWE, PWE+CE, and PWE+SFE) were statistically significantly different from the baseline (ie, RIWE), where random embeddings were used. This means that pretraining embeddings on the target domain (ie, discharge summaries from MIMIC-III) helped in comparison with the random initialization of WEs.

**Table 5:** Post-hoc analysis of variance (ANOVA) of the named entity recognition models: *P* values of two-tailed paired t tests for each pair of models.^a^

Named entity recognition	PWE^b^, *P* value	PWE+CE^c^, *P* value	PWE+SFE^d^, *P* value
RIWE^e^	<.001	<.001	<.001
PWE	N/A^f^	.94	.99
PWE+CE	N/A	N/A	.95

^a^RIWE is significantly worse than the rest of the models. At the same time, there is no statistically significant difference between PWE, PWE+CE, and PWE+SFE.

^b^PWE: pretrained word embeddings.

^c^CE: character embedding.

^d^SFE: semantic-feature embeddings.

^e^RIWE: randomly initialized word embeddings.

^f^N/A: not applicable.

### RE Models

Table 6 shows the performances of the RE models using the gold-standard entities, whereas Table 7 shows the performances of the RE model using the output from the NER models (end-to-end). Using the gold-standard entities and using the output from the best NER model (end-to-end), we achieved micro F-scores of 0.927 for rules and 0.855 for (PWE+CE)+rules, respectively. Thus, the traditional rule-based method performed surprisingly well relative to the context-aware LSTM for this task. Relations between form and frequency to drugs are examples of such success: there was at least a 4% improvement in F-score over the LSTM model. The microaverage F-score for the end-to-end task was notably lower than that for the NER tasks and RE using gold-standard entities. This was expected because prediction in the end-to-end compounded the errors in both the NER and RE steps. A major factor behind the low score is the reasons-drug relation type, which was often not recognized because the NER did not recognize the reason attribute. However, the prediction of this relation itself (ie, reason-drug) is also challenging, as evidenced by the F-score of 0.734 in the RE task (rules) on the gold-standard entities. This might be because the text span between 2 entities in this relation is often relatively long; thus, none of the methods explored in this study could capture this.

**Table 6:** Evaluation results of the relation extraction models (using gold-standard entities) on the test set (lenient evaluation).

Relation type	LSTM^a^			Rules^b^		
	Precision	Recall	F-score	Precision	Recall	F-score
Strength-drug	0.973	0.961	0.967	0.963	0.988	*0.975^c^*
Dosage-drug	0.963	0.958	0.961	0.956	0.976	*0.966*
Duration-drug	0.909	0.892	0.901	0.942	0.880	*0.910*
Frequency-drug	0.962	0.904	0.932	0.964	0.988	*0.975*
Form-drug	0.982	0.918	0.949	0.970	0.992	*0.981*
Route-drug	0.958	0.934	0.946	0.962	0.972	*0.967*
Reason-drug	0.741	0.830	*0.783*	0.767	0.704	0.734
Micro	0.922	0.913	0.918	0.937	0.917	*0.927*
Macro	0.914	0.910	0.909	0.935	0.902	*0.917*

^a^LSTM: long-short term memory method.

^b^Rules: rule-based method.

^c^The best results for each metric are italicized.

**Table 7:** Evaluation results of the end-to-end models (ie, output from the best-performing named entity recognition and relation extraction models) on the test set (lenient evaluation).

Relation type	RIWE^a^+rules				PWE^b^+rules				(PWE+CE)^c^+rules				(PWE+SFE)^d^+rules		
	Precision	Recall	F-score	Precision		Recall	F-score	Precision		Recall	F-score	Precision		Recall	F-score
Strength-drug	0.919	0.914	0.917	0.952		0.943	0.947	0.948		0.950	0.949	0.948		0.964	*0.956^e^*
Dosage-drug	0.848	0.853	0.851	0.890		0.888	0.889	0.892		0.884	0.888	0.894		0.897	*0.895*
Duration-drug	0.837	0.615	0.709	0.842		0.662	0.741	0.889		0.617	0.729	0.860		0.678	*0.759*
Frequency-drug	0.878	0.874	0.876	0.931		0.919	0.925	0.949		0.902	0.925	0.934		0.947	*0.940*
Form-drug	0.894	0.888	0.891	0.939		0.915	0.927	0.944		0.919	0.931	0.959		0.920	*0.939*
Route-drug	0.885	0.866	0.875	0.924		0.895	0.909	0.919		0.904	0.911	0.920		0.908	*0.914*
Reason-drug	0.635	0.333	0.437	0.702		0.371	0.485	0.744		0.343	0.470	0.503		0.472	*0.487*
Micro	0.865	0.770	0.815	0.909		0.802	0.852	0.918		0.797	*0.855*	0.871		0.830	0.850
Macro	0.859	0.733	0.784	0.902		0.770	0.824	0.918		0.765	*0.824*	0.849		0.801	0.821

^a^RIWE: bidirectional long-short term memory with conditional random fields with random word embeddings.

^b^PWE: bidirectional long-short term memory with conditional random fields with pretrained word embeddings.

^c^PWE+CE: bidirectional long-short term memory with conditional random fields with pretrained word embeddings and character embeddings.

^d^PWE+SFE: bidirectional long-short term memory with conditional random fields with pretrained word embeddings and semantic-feature embeddings.

^e^The best results for each metric are italicized.

The statistical significance test for the RE task showed that the differences between the LSTM and rule-based models were insignificant (*P*=.41). For the end-to-end task, similar to the NER task, there was no statistically significant difference between any of the models (PWE, PWE+CE, and PWE+SFE); however, the 3 models were statistically significantly different from the RIWE (Table 8). Accordingly, the best-performed end-to-end system, (PWE+CE)+rules, was chosen for our DrugEx system.

**Table 8:** Post-hoc analysis of variance (ANOVA) of the end-to-end models: *P* values of two-tailed paired *t* tests for each pair of models.

End-to-end models	PWE^a^+rules, *P* value	(PWE+CE^b^)+rules, *P* value	(PWE+SFE^c^)+rules, *P* value
RIWE^d^+rules	.01	.01	.03
PWE+rules	N/A^e^	.99	.99
(PWE+CE)+rules	N/A	N/A	.99

^a^PWE: pretrained word embeddings.

^b^CE: character embedding.

^c^SFE: semantic-feature embeddings.

^d^RIWE: randomly initialized word embeddings.

^e^N/A: not applicable.

## Discussion

### Principal Findings

The models explored in this study demonstrated high F-scores of 0.921 for NER, 0.927 for RE, and 0.855 for the end-to-end approach. The overall highest F-scores (achieved by different teams) in the n2c2 challenge in the NER, RE, and end-to-end tasks were 0.942, 0.963, and 0.891, respectively [13]. The top-ranked NER used a BiLSTM-CRF with ELMo language model [45], CFEs, and normalized section titles as features. The top-ranked RE and end-to-end tasks used a joint concept-relation extraction system that uses 2 layers of BiLSTM-CRFs [58].

The results for our NER models showed that PWE+CE had the highest classification efficiency, followed by PWE and PWE+SFE, which had similar scores among themselves and above the baseline. RE models’ results showed that the rule-based method achieved significantly higher accuracy than the context-aware LSTM for most relation types. Interestingly, the LSTM model performed notably better in the reason-drug relations, which were missed more than all other relation types.

We observed that external resources (ie, SFEs) contributed to the attribute extraction. Presumably, plentiful labeled data already available and complementary information from these external resources appear to have been helpful for performance. Nevertheless, simpler methods, such as PWE and rule-based methods, can match these sophisticated and expensive methods.

### Error Analysis

We further analyzed false positives and false negatives from the NER to obtain deeper insights into the common classification errors. Note that the focus in the error analysis was on the NER only, as it appears to be the main factor of the relatively low F-score in RE.

To gain an insight into where errors are made and how models can be improved, we manually reviewed false negatives (entities identified in the gold standard but incorrectly rejected, ie, missed, by the models) and false positives (entities identified by the models when they are not in the gold standard) in the best-performing model. Errors were then grouped into different categories based on their causes, including (1) context error: when an entity is captured as one of the drug-related attributes, although it is not, or when an entity is missing because of the context; (2) type error: when an attribute is extracted but with an incorrect annotation type; and (3) gold-standard error: possible error in the gold standard. We also generated a confusion matrix to subdivide the errors made by the method based on which type of mistake was made.

Context error was a major category of errors. These mostly resulted from previously unseen information (eg, “He was given a loading dose of amiodarone,” where the dosage *loading dose* was missed), atypical expression formats (eg, “One (1) Tablet,” where dosage *one (1)* was missing because of the parentheses), and abbreviations (eg, “Dig level 2.1,” where drug *dig*—which should be *digoxin*—was missed). Context errors may also result from the complexity of language expressions; for example, *200 units* in the phrase “was started on a 7d course of DRUG 200 units daily” could be a dosage when considered as a single phrase, or it could be 2 concepts: 200 (a strength) and unit (a form). Gold annotation preferred the latter, whereas our method identified the former.

**Figure 5:**
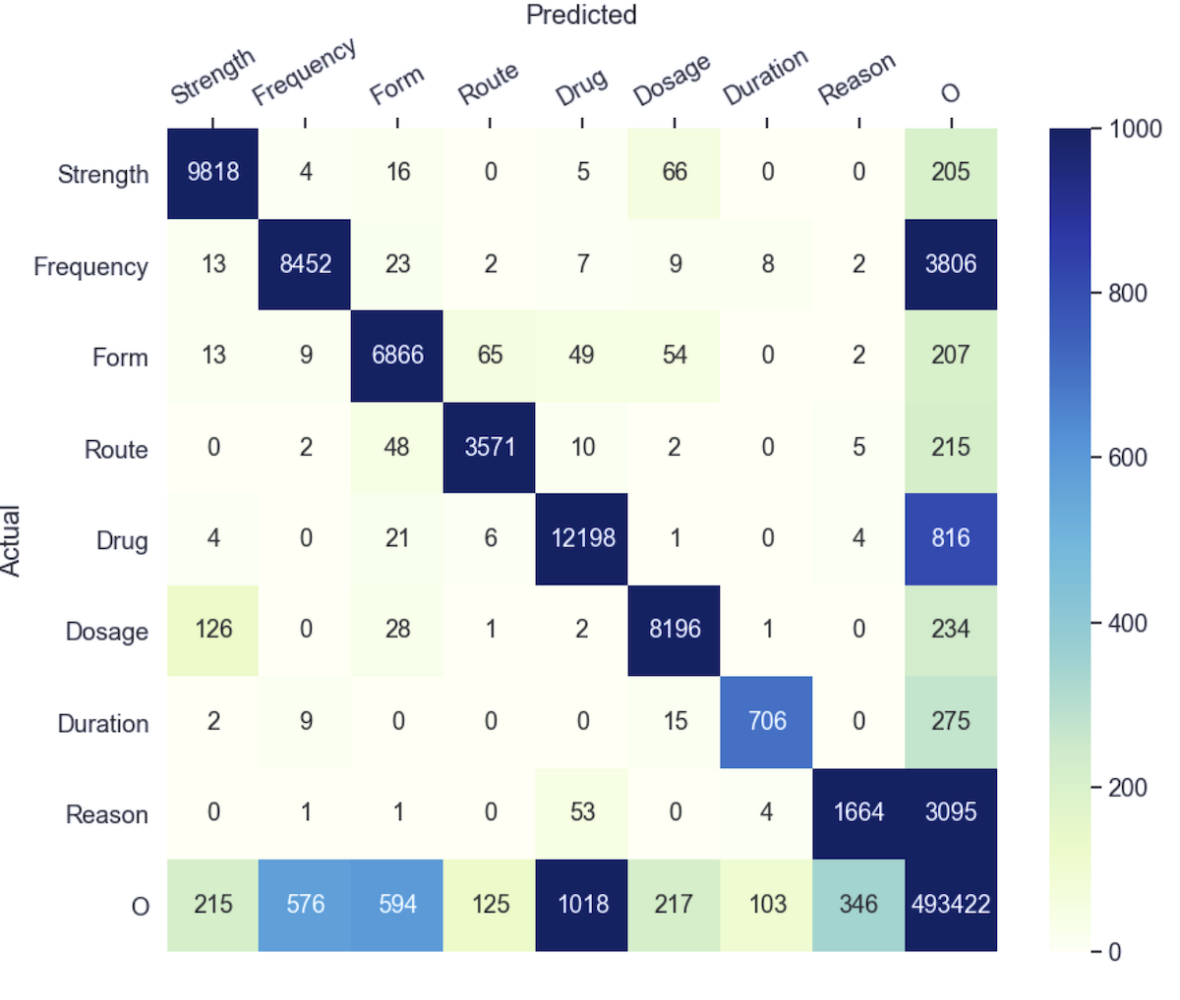
Confusion matrix (token-level) from the output of bidirectional long-short term memory with conditional random field (with pretrained word embeddings and character embeddings) on the National NLP Clinical Challenges test set. The diagonal entries indicate labels that were correctly predicted, and the off-diagonal entries indicate errors. The total number of errors (sum of off-diagonal cells) was 693.

Another interesting cause of error is the ambiguity between attributes, where an attribute is recognized, but the type is incorrect. Figure 5 presents the confusion matrix for the BiLSTM-CRF (PWE+CE) and indicates how often each entity is predicted. The confusion of dosage for strength and strength for dosage is the most frequent type of error, accounting for 28% ([66+126]/693) of the errors. The following example illustrates this type of error: “Meropenem 500 mg Intravenous every eight (8) hours.” The dosage *500 mg* is wrongly predicted as strength; usually, the mg unit is associated with strength. The substitution of dosage for strength is a common error, and these entities are often mislabeled as each other—both are often numeric quantities and used in similar contexts. A common solution for this issue is to merge these 2 types into 1 annotation type [59]. However, extracting them separately may be important for some applications.

The second most frequent type of this error, which accounts for 16% ([48+65]/693) of the errors, is the confusion of form for route and route for form. These entities are often annotated as the gold standard in various ways. For example, the word *injection* is sometimes annotated as a form and sometimes as a route; in the training set, it is annotated as a form 68 times and as a route 53 times, which makes learning from these examples challenging.

The confusion of drugs with general words is one of the other sources of error. We found that there were several causes of this confusion among drug names. These include (1) generic drug names (eg, *glucose, IVF, blood, D5W,* and *chemo*) corresponding to prescribed medications but not occurring in expected contexts; (2) words such as *pressor*, *fluids*, *agents,* or *medication* that may be considered to be underspecified, but should be extracted, at least in this data set; (3) some classes of drugs (eg, *antiinflammatory drugs* and *hypertension medications*) missing in the training sources; (4) new drug names that did not occur within an expected context or semantic patterns (eg, *Dig level 2.5*), so they were not extracted by the NER methods; and (5) abbreviations (eg, *aspirin325* and *ABX*).

The analysis also showed a few potential omissions and inconsistencies in human annotations. Gold-standard errors fall into 2 different categories: missing in the gold standard and potential problems in gold standards. The more common error in this category is missing in the gold standard, where the method annotates entities that are not annotated in the data set. For example, *four weeks* in the phrase, “adding DRUG cover for the first four weeks of treatment,” is not annotated as a duration in the gold standard, whereas it appears to be a potentially correct attribute. Inconsistency may also appear in annotation spans; for example, dosage or strength, and form were annotated separately sometimes and jointly in others.

### Conclusions

In this study, we constructed an end-to-end system (DrugEx) composed of bidirectional LSTM, CRF, and rule-based methods for extracting drug-related information from free-text discharge summaries. We studied various token representations (ie, WE, CE, and SFE) for extracting drug attributes from free-text discharge summaries. We also proposed a rule-based method for relations between drugs and attributes and compared this method with a context-aware deep learning method. The results showed that the proposed system can be used successfully for extracting and linking drug attributes in discharge summaries, although some attributes (ie, reason and duration) are still challenging. The results also showed that domain-tailored embeddings (ie, PWE) perform better than random embeddings (RIWE) in this task. Concatenating PWE with CE or SE achieved a comparable overall performance when compared between themselves. NER (PWE+CE) ranked best in F-score among other proposed models; however, NER (PWE+SE) performed better on some individual entity types, especially frequency, duration, and reason. Semantic embeddings also yielded a better balance between precision and recall. However, a simpler method (eg, WE and CE) can match these sophisticated and expensive methods. Incorporating external knowledge (eg, of a drug’s reason, proposed treatment, and a drug’s reactions) and incorporating a larger context may improve performance.

Concerning RE, the rule-based method achieved higher accuracy than the context-aware LSTM for most relations. Interestingly, the LSTM model performs notably better on some of the most challenging relations (eg, reason-drug).

In future work, we aim to investigate contextual embeddings, such as ELMo and BERT, which have been proven to provide considerable improvements in other tasks that include complex language structures, ambiguous word use, and unseen words in training. We also consider assessing the performance and transferability of the models across different biomedical data sets and tasks.

Finally, the medication NER and RE tasks are important not only from a research perspective but also because they have applications as steps in practical information extraction pipelines. The current level of performance indicates that these models should be good enough for large-scale statistical and epidemiological studies. However, applications that require patient-specific information may need NER systems with even higher recall and precision, ensemble and multiple-step systems (ie, systems that combine the output of multiple classifiers), or be subject to semiautomated verification.

## Abbreviations

ADE: adverse drug event
BERT: Bidirectional Encoder Representations from Transformers
BiLSTM: bidirectional long-short term memory
BiLSTM-CRF: bidirectional long-short term memory with conditional random field
BIOES: Begin, Inside, Outside, End, Single
CE: character embedding
CLAMP: Clinical Language Annotation, Modeling, and Processing Toolkit
CRF: conditional random field
cTAKES: Clinical Text Analysis and Knowledge Extraction System
EHR: electronic health record
ELMo: Embeddings from Language Models
LSTM: long-short term memory
MIMIC-III: Medical Information Mart for Intensive Care III
n2c2: National NLP Clinical Challenges
NER: named entity recognition
NLP: natural language processing
PWE: pretrained word embedding
RE: relation extraction
RIWE: randomly initialized word embedding
SFE: semantic-feature embedding
WE: word embedding
